# Perception Regarding the NICE Guideline on Antibiotic Prophylaxis against Infective Endocarditis Following Dental Procedures: A Cross-Sectional Study

**DOI:** 10.3390/antibiotics12040696

**Published:** 2023-04-02

**Authors:** Siti Salmiah Mohd Yunus, Syed Nabil, Muhd Fazlynizam Rashdi, Abd Jabar Nazimi, Rifqah Nordin, Huann Lan Tan, Oteh Maskon, Hamat H. Che Hassan, Tzar Mohd Nizam Khaithir, Aznida Firzah Abdul Aziz, Yee Guan Ng, Ridwan Yeop Ismail, Roszalina Ramli

**Affiliations:** 1 Department of Oral & Maxillofacial Surgery, Faculty of Dentistry, Universiti Kebangsaan Malaysia (UKM), Kuala Lumpur 50300, Malaysia; 2 Department of Oral & Maxillofacial Surgery, Hospital Canselor Tuanku Muhriz (HCTM), Universiti Kebangsaan Malaysia, Kuala Lumpur 56000, Malaysia; 3 Department of Medicine (Cardiology Unit), Faculty of Medicine, Universiti Kebangsaan Malaysia (UKM), Kuala Lumpur 56000, Malaysia; 4 Department of Microbiology and Immunology, Faculty of Medicine, Universiti Kebangsaan Malaysia (UKM), Kuala Lumpur 56000, Malaysia; 5 Department of Family Medicine, Faculty of Medicine, Universiti Kebangsaan Malaysia (UKM), Kuala Lumpur 56000, Malaysia; 6 Department of Environmental Occupational Health, Faculty of Medicine and Health Sciences, Universiti Putra Malaysia (UPM), Serdang 43400, Malaysia; 7 Department of Oral & Maxillofacial Surgery, Hospital Tuanku Fauziah, Kangar 01000, Malaysia

**Keywords:** endocarditis, practice guideline, antibiotic prophylaxis, dental care, heart disease

## Abstract

This study explores the opinions of Malaysian clinical specialists on the antibiotic prophylaxis against infective endocarditis (IE) as described in the 2008 National Institute for Health and Care Excellence (NICE) guideline. This cross-sectional study was performed from September 2017 to March 2019. The self-administered questionnaire comprised two sections: background information of the specialists and their opinions on the NICE guideline. The questionnaire was distributed to 794 potential participants, and 277 responded (response rate of 34.9%). In general, 49.8% of the respondents believed that clinicians should adhere to the guideline, although the majority of oral and maxillofacial surgeons (54.5%) actually disagreed with this view. The dental procedures that were perceived as presented moderate-to-high risk for IE were minor surgery for an impacted tooth with a recent episode of infection, dental implant surgery, periodontal surgery and dental extraction in patients with poor oral hygiene. The cardiac conditions that were strongly recommended for antibiotic prophylaxis were severe mitral valve stenosis or regurgitation and previous IE. Less than half of Malaysian clinical specialists agreed with the changes in the 2008 NICE guideline, contributing to their insistence that antibiotic prophylaxis is still needed for high-risk cardiac conditions and selected invasive dental procedures.

## 1. Introduction

Infective endocarditis (IE) is a potentially life-threatening infection involving the heart valves or the endocardium. It is most often related to congenital or acquired cardiac defects. High-risk individuals susceptible to IE following dental procedures include patients with prosthetic heart valves, previous IE and untreated congenital heart disease [1]. During invasive dental procedures, especially in a poor oral hygiene context, bleeding from the oral mucosa may allow for oral microflora such as streptococci to enter the bloodstream. Any person with a damaged heart valve endothelium is more prone to bacterial valvular colonisation [2]. Considering this risk, antibiotic prophylaxis (AP) has been recommended particularly to high-risk patients prior to invasive dental procedures [3].

The use of AP was likely first proposed in the 1940s and the guidelines for its use have been revised many times since then [3]. In March 2008, the National Institute for Health and Care Excellence (NICE) issued a significant change from its previous editions, indicating that AP is not recommended for people undergoing dental procedures [4]. The guideline was updated in 2015 and amended in 2016 [4]. Around the same period, the American Heart Association (AHA) (in 2007) [5] and the European Society of Cardiology (ESC) (in 2009) [6] revised their guidelines recommending AP for high-risk patients. The justification for the changes in the NICE guideline was the concern about the possible adverse effects of AP, including the potentially fatal anaphylaxis reaction [4]. Moreover, public health implications of AP, namely the rise in antibiotic resistance and the cost ineffectiveness of such measures, were also mentioned [4]. The emergence of multidrug-resistant bacterial infection due to inappropriate use of antibiotics poses a grave threat to public health [7]. The ineffective use of AP can also contribute to wastage of the already limited health care funds. There are, however, opposing views on these matters. Opatowski et al. [8] suggest that using a single high dose of AP minimises the risk of resistance. Meanwhile, Franklin et al. [9] found that AP for IE is in fact cost-effective, even in non-high-risk patients.

The 2008 NICE guideline has invited a significant amount of confusion and criticism, particularly from clinicians who either directly or indirectly manage patients with IE risk. Moreover, the AHA [5] and the ESC [6] guidelines did not make such a drastic change. In the UK, despite the new NICE guidelines, the majority of cardiologists still insist on prescribing antibiotics for high-risk individuals [10]. Dental practitioners, on the other hand, have been more accepting to the change in the recommendation [10]. Another survey outside the UK found that cardiologists and dental practitioners prefer the AHA guideline over the NICE guideline [11].

Besides cardiologists and oral and maxillofacial surgeons (OMFSs), there are other specialists involved in the management of patients at risk of IE. Family medicine specialists (FMSs) may be the first to attend to the patients prior to referral to cardiologists. Microbiologists and forensic pathologists are also engaged in the process of providing the IE diagnosis, albeit not in clinic settings. Thus, their opinions on this subject are worth exploring. The purpose of this study was to explore the opinion of clinical specialists regarding the 2008 NICE clinical guideline (updated in 2015) recommendation of AP for IE. We also attempted to gain insights from clinicians regarding some dental procedures and cardiac conditions.

## 2. Materials and Methods

### 2.1. Study Design, Period, Settings and Participants

This cross-sectional study used a questionnaire. The potential participants were approached between September 2017 and March 2019. The study protocol was approved by the Universiti Kebangsaan Malaysia Research Ethics Committee (UKM PPI/111/8/JEP-2017-202) and the National Committee for Clinical Research (NCCR) of Malaysia (NMRR-17-913-33977(IIR)). By completing and submitting the questionnaire, the respondents indicated their consent to participate in this study. The respondents were from public and private tertiary hospitals throughout Malaysia. The National Heart Association (NHA) was approached to increase participation from cardiologists.

The participants included specialists who had experience working with patients with IE. This included cardiologists, OMFSs, FMSs, microbiologists and forensic pathologists. OMFSs were selected to represent the dental practitioners because the oral and maxillofacial surgery clinics in hospitals are the routine referral centres for managing dental diseases among patients at risk for IE in Malaysia. The names and contact numbers of these specialties were obtained from the National Specialist Register (NSR) and the Malaysian Association of Oral and Maxillofacial Surgeons (MAOMS) websites. The NSR and MAOMS represent the country’s databases for medical specialists and OMFSs. The registers contain information about the specialists and their disciplines/specialties, qualifications and places of practice.

### 2.2. Study Tool: Questionnaire

The questionnaire was developed based on the content of the 2008 NICE guideline (updated in 2015).

#### 2.2.1. Content of the Questionnaire

The structured questionnaire included two main sections: section one on demographics and basic information (six questions) and section two on the participants’ feedbacks on AP for IE. Section two consisted of three subsections (Table 1). Any incomplete questionnaires were excluded from the analysis.

#### 2.2.2. Pre-Test

The questionnaire was pre-tested to evaluate its clarity and reliability. The pre-test was performed via an email invitation with link to the questionnaire sent to respondents within our hospital from each of the specialties involved in this study.

### 2.3. Distribution of the Questionnaire

The questionnaire was distributed via multiple methods to maximise participation. It was mostly distributed to the participants via email. The questionnaire was provided online via a Google survey form (http://doc.google.com/forms, accessed on 31 December 2019). Other methods included the WhatsApp Messenger and hand delivery. Postage delivery was not recommended as advised by the specialty reps. In addition, face-to-face appointments were also attempted.

### 2.4. Statistical Analysis

The data were analysed using the Predictive Analytics Software (PASW; formerly SPSS) Statistics Version 18.0 (SPSS Inc., Chicago, IL, USA). Descriptive statistics of the study factors, characteristics and outcomes of interest are shown as frequencies, percentages, mean, standard deviation (SD) or median and interquartile range (IQR). Pearson’s chi-squared test for a multi-way contingency table was performed to evaluate the association between the questions and the specialists’ responses. Fisher’s exact test was used if >20% of the expected cell counts were less than 5. The level of significance was set at 5% for all tests.

## 3. Results

### 3.1. Participant Characteristics

The questionnaire was distributed to 794 potential participants identified from the NSR. Of the 794 participants, 380 were cardiologists, 94 were the OMFSs, 321 were FMSs, 52 were microbiologists and 47 were forensic pathologists. The total number of complete responses from all specialities were 277, and the majority of the participants were from the Ministry of Health hospitals (*n* = 181). This resulted in a total response rate of 34.9%. Fifteen participants (1.9%) attempted to but did not complete the questionnaire.

Among the specialties, forensic pathologists had the highest response rate (100%), followed by OMFSs (59%). The response rates from the microbiologists, FMSs and cardiologists were 54%, 34% and 10%, respectively. The demographics of the participants are provided in Table 2. Most of the specialists who took part in this survey were <40 years old (53.8%). 

The age range was between 27 and 68 years old and the median (IQR) was 39 (11) years. The male-to-female ratio was 1:1.42. Most worked at Ministry of Health hospitals (65.3%). Overall, 72.9% had ≤10 years of working experience while 27.1% had >10 years of working experience. They had been practicing as a specialist for 1.0–30 years (median = 5, IQR = 10).

### 3.2. Reliability of the Questionnaire

Thirty-one participants took part in the pre-test: five cardiologists, four OMFSs, fifteen microbiologists and seven forensic pathologists. Cronbach’s α was 0.67, which is considered acceptable. 

### 3.3. Awareness and Adherence to the 2008 NICE Guideline (Updated in 2015)

Almost half of the participants were aware of the 2008 NICE guideline when it was first released (Table 3). Two specialties—cardiology and oral and maxillofacial surgery—that were directly involved in treating patients with the risk for IE had the highest awareness, 69.2% and 78.2%, respectively. Considering all respondents, 48.7% were aware, 39.7% were unaware and 11.6% were unsure (*p* < 0.001) (Table 4). Forty-two percent of the participants agreed with the guideline change, 30% disagreed and 27% were unsure (*p* < 0.001) (Table 4). Among the specialties, OMFSs exhibited more disagreement (54.5%) to the change in the guideline while among the cardiologists, 48.7% recommended following the 2008 NICE guideline and 38.5% were against it. Considering all the respondents, 49.8% agreed, 32.1% disagreed and 18.1% were unsure (*p* < 0.001) (Table 4).

We also evaluated the association between the above responses and years of practising as a specialist, age and whether the specialist was based in a government or a private hospital; all results were not significant (*p* > 0.05). Only gender showed a significant association with the responses.

(i) Question 2.1

Gender (39.7% (no) vs. 48.7% (yes) vs. 11.6% (unsure), *p* = 0.02);

(ii) Question 2.2

Gender (30.3% (no) vs. 42.2% (yes) vs. 27.4% (unsure), *p* < 0.001);

(iii) Question 2.3

Gender (32.1% (no) vs. 49.8% (yes) vs. 18.1% (unsure), *p* = 0.006).

### 3.4. Risk Levels of Selected Dental Procedures

This part of the questionnaire aimed to evaluate the responses from the specialists about the risk of IE for different types of dental treatment (Table 5). Overall, the majority of the participants agreed that the dental procedures below posed a low risk for IE (Table 6).

(i) Routine dental fillings (71.5% (low risk) vs. 21.3% (medium–high risk) vs. 7.2% (unsure); *p* < 0.001);
(ii) Scaling and polishing (60.3% (low risk) vs. 33.9% (medium–high risk) vs. 5.8% (unsure); *p* = 0.02).

The specialists perceived other listed dental procedures as having moderate to high risk for IE (Table 6).

(i) Periodontal surgery (74.4% (medium-high risk) vs. 21.3% (low risk) vs. 4.3% (unsure); *p* = 0.02);
(ii) Extraction of tooth/teeth (68.6% (medium-high risk) vs. 28.2% (low risk) vs. 3.2% (unsure); *p* = 0.01);
(iii) Minor surgery for an impacted tooth with a recent episode of infection (83.8% (medium-high risk) vs. 11.6% (low risk) vs. 4.7% (unsure); *p* = 0.08);
(iv) Root canal treatment (69.3% (medium-high risk) vs. 25.3% (low risk) vs. 5.4% (unsure); *p* < 0.001);
(v) Dental implant surgery (79.8% (medium-high risk) vs. 14.8% (low risk) vs. 5.4% (unsure); *p* = 0.06);
(vi) Dental extraction in patients with poor oral hygiene (84.8% (medium-high risk) vs. 11.6% (low risk) vs. 3.6% (unsure); *p* = 0.30).

Among the listed dental procedures, the respondents overwhelmingly considered minor surgery for an impacted tooth with a recent episode of infection, dental implant surgery and dental extraction in patients with poor oral hygiene as representing a medium-to-high risk for IE.

### 3.5. Requirement for AP Prior to Dental Procedures in Selected Cardiac Conditions

The majority of the specialists recommended AP for all cardiac conditions except for mild mitral valve stenosis/regurgitation and hypertrophic cardiomyopathy (HCM) (Table 7). The following were the percentages of response for cardiac conditions that do not require AP (Table 8).

(i) Mild mitral valve stenosis/regurgitation (52.3% recommended no AP vs. 37.9% recommended AP vs. 9.7% were unsure, *p* < 0.001);
(ii) HCM (44.0% recommended no AP vs. 42.2% recommended AP vs. 13.7% were unsure, *p* = 0.03).

All other cardiac conditions received various percentages of agreement for AP (Table 8).

(i) Moderate mitral valve stenosis/regurgitation (65.0% recommended AP vs. 28.5% recommended no AP vs. 6.5% were unsure, *p* = 0.01);
(ii) Severe mitral stenosis/regurgitation (81.9% recommended AP vs. 10.8% recommended no AP vs. 7.2% were unsure, *p* = 0.37);
(iii) Previous IE (88.1% recommended AP vs. 6.5% recommended no AP vs. 5.4% were unsure, *p* = 0.26);
(iv) Structural congenital heart disease (SCHD) (66.4% recommended AP vs. 25.3% recommended no AP vs. 8.3% were unsure, *p* = 0.03).

Among the cardiac conditions, severe mitral valve stenosis/regurgitation and previous IE received overwhelming recommendation for AP.

## 4. Discussion

There are relatively scare epidemiological data regarding IE in developing countries, including Malaysia. It is clear that the epidemiology of IE in developing countries differs from that in developed countries. Data from the UK revealed that the risk of developing IE is highest in those with a previous history of IE and those with prosthetic or repaired valves [12]. At the same time, findings from Laos, a developing country, indicated that IE is more associated with rheumatic heart disease (RHD), followed by congenital heart diseases [13]. A recent study in Malaysia similarly found that RHD represented the most common predisposing factor among the reported IE cases [14]. With a mean incidence of 31 IE cases per 100,000 adult admissions, the incidence in Malaysia is higher than in the developed countries and the trend is increasing [14].

There are guidelines from NICE, the AHA and the ESC emphasising IE prevention following dental procedures in at-risk patients. When the NICE guideline was first published in 2008, it created split opinions among clinicians particularly due to the statement that “antibiotic prophylaxis is not recommended”, a drastic shift from the previous guideline recommendation. The 2008 NICE guideline 2008 was updated in 2015 and amended in 2016 by adding the word “routinely” to the statement. It now reads “Antibiotic prophylaxis against infective endocarditis is not recommended routinely for people undergoing dental procedures” [15]. Many dental practitioners felt that the 2008 version of the guideline appeared to prohibit any AP [15]. The addition of such wording allows for some leeway for individual cases were the risk of IE to the patient is thought to be elevated [15].

In this study, OMFSs and cardiologists displayed a high awareness of the 2008 NICE guideline (updated in 2015). This is an expected outcome because both specialties are directly engaged in this clinical scenario. Although the other specialities may not have been aware when the guideline was first released, the majority of them provided an opinion about whether they agreed or disagreed with the changes made. This finding suggests that awareness of the guideline has improved over time even among specialists who do not directly treat these patients. We found that almost half of the participants agreed with the change made in the guideline. OMFSs mostly did not agree to adhere to the recommendation. This finding is intriguing because OMFSs and dentists in general are unable to observe the consequences of administering AP—in other words, they cannot determine whether they have prevented or caused IE by either prescribing or not prescribing AP. It is possible that OMFSs are being pressured to prescribe AP from both the patient and their treating cardiologist [10]. Furthermore OMFSs, particularly in Malaysia, may also be influenced by the fact that other guidelines do not share the same recommendation [16]. An interesting finding that we found was that there was statistically significant association between gender and the response for the aforementioned questions. This association is likely due to the overrepresentation of males among cardiologists and OMFSs, who were mostly aware of the changes and agreed with them, but did not comply with the recommendations.

In Malaysia, the Ministry of Health published its local guideline in 2017, the Clinical Practice Guidelines for the Prevention, Diagnosis and Management of Infective Endocarditis [17]. The notable difference in the Malaysian guideline is a more exhaustive list of at-risk patients compared with other guidelines. This guideline affirmed its stand on the AP requirement after considering the local context considering that the situation in Malaysia is different from the UK, US or Europe. For example, the most common predisposing factor for IE in Malaysia is RHD. The level of oral hygiene in the Malaysian population is also different from the patients from Western countries [18]. Hence, when a local guideline is available, it should be the main reference and other guidelines should act as additional references. Discussion with the referring cardiologist is also considered to be good practice.

We attempted to paint an overall picture of the practices and opinions regarding IE with dental and medical specialists. We also involved other experts who are indirectly involved in managing IE cases. Although the medical specialists may not be very well versed with dental procedures, their recommendation to provide AP for the medium-to-high risk procedures was rather acceptable. Other studies have also sought out the opinions of various specialists on the use of AP against IE. Dayer et al. [10] included microbiologists and infectious disease specialists alongside dental specialists, cardiothoracic surgeons and cardiologists in their survey, while Soheilipour et al. [19] (UK) and Rı’orda’in and McCreary [11] (Ireland) included cardiologists and dentists in their studies. In addition, Alhuzaimi et al. [20] assessed the knowledge and practice attitudes of cardiologists and cardiothoracic surgeons towards the IE AP guidelines in Saudi Arabia.

Our finding according to which most specialists may prescribe AP for high-risk cases is in good agreement with the results of previously published studies. Dayer et al. [10] found that clinicians gave the opinion that patients with a prosthetic heart valve or previous history of IE should receive AP. On the contrary, Alhuzaimi et al. [20] reported an overprescribing attitude for low-risk cardiac lesions. Meanwhile, many studies have shown that dentists are still uncertain about prescribing AP [10,11,19]. Many still seek advice from cardiologists instead of referring to the guidelines before prescribing AP to patients [10,11,19]. This study showed that most microbiologists recommend adhering to the NICE guidelines, a finding that concurs with the Saudi Arabian study [20].

There are implications to overprescribing antibiotics. Adverse effects of antibiotics, such as angioedema, anaphylaxis and antibiotic resistance, are among the main reasons for the change in the 2008 NICE guideline. Evidence from the UK has shown a fatal adverse drug reaction rate for clindamycin of 13 per 1 million prescriptions; on the other hand, for amoxicillin AP, the fatal reaction was 0 per nearly 3 million prescriptions [21]. Moreover, high rates of amoxycillin and clindamycin resistance have been reported [22]. However, there are antibiotics with low antibiotic resistance and high susceptibility of bacteria, such as the amoxycillin with clavulanic acid and moxifloxacin, that should be considered as AP [22].

There is a rather large number of conflicting opinions regarding cardiac conditions that require AP and the available guidelines define high-risk groups differently [3]. Nevertheless, most guidelines agree that prosthetic valves, previous IE and untreated congenital cyanotic heart disease have an elevated risk for the development of IE [1,4,17,23]. Of note, HCM is not mentioned in the AHA or ESC guidelines [1,4,23]. In this study, HCM was the only condition with an almost equal percentage of participants stating AP is required or not required. It is also the cardiac condition with the highest “unsure” response. This confusion regarding HCM is likely related to the difference between the three major guidelines on this condition. Our findings concur with another study that showed that acquired valvular heart disease and HCM carry less risk for developing IE, and hence do not require AP [24]. Some authors, however, still suggest AP for HCM due to its high mortality rate [25]. The contradictory opinions may lead to AP being prescribed inappropriately [26]. In a large Spanish cohort study, Dominguez et al. [25] recommended that patients with HCM with or without left ventricular outflow tract obstruction could benefit from AP against IE. They also showed that dental procedures and streptococcal infection are among the predominant predisposing factors in patients with HCM and IE [25].

Dental procedures with manipulation of the gingiva or periapical tissue and oral mucosa perforation carries the risk of causing IE [1]. The guidelines emphasise that, besides the type of procedure, oral and dental hygiene are an important factor in preventing IE among the population at risk. Therefore, the oral hygiene status of a patient should be considered when deciding on the need for AP [1,4]. This is one of the efforts highlighted recently in preventing IE [1,4,5]. The oral hygiene status of the Malaysian population could be considered poor based on the data from the Malaysia’s National Oral Health Survey of Adults 2010: 88.9% of adults had dental caries, 94% had periodontal disease and 98.3% needed oral health care but less than one-third of adults utilised oral health care services in the past year [18]. It is important to note that even though there is higher risk of bacteraemia during tooth brushing in a poor oral hygiene environment, there is also a risk of bacteraemia following dental extractions regardless of the oral hygiene status [27]. Overall, the invasiveness of the dental procedure and the oral hygiene status should both be considered when deciding on whether to administer AP. We found that the respondents from all of the selected specialties had at least a common understanding regarding the risk of IE from different procedures, with dental extraction in a poor oral hygiene environment deemed the most high-risk procedure and dental filling as the least risky procedure.

The biggest limitation of this study is related to small percentage of participation from the cardiologists. We made various efforts to improve their participation. Our team approached the NHA during the initial stage to engage all cardiologists in Malaysia through the NHA’s official email list. However, the email response was very low despite multiple emails sent by the NHA. Subsequently, we initiated a strategy to participate in the NHA scientific meeting to distribute the questionnaire. Logistically, this strategy should have yielded many responses. However, our request was denied. Thus, we visited all the cardiology centres to distribute the questionnaires by hand; this effort also did not generate many responses due to their hectic work schedules. Hence, the limitation of this study is that, although we invited nearly 400 cardiologists to complete the questionnaire, only 39 responded (a 10.3% response rate). The low participation from cardiologists has also been reported in other studies [19].

Our study has several strengths. First, it encompasses several specialties that had not been previously explored, offering insights from specialists whose opinions on the topic were crucial but had not been collectively sought before. Second, while previous studies have focused on the opinions of dentists regarding the guidelines, we took a unique approach by seeking the perspective of OMFSs, who are qualified personnel with dental experience based in hospitals and are more likely to encounter such clinical situations frequently compared with their office-based counterparts. Third, because we conducted this study in Malaysia, it provides insights into how the guidelines from an internationally respected organisation affect the opinions and practices of clinicians working in developing countries.

## 5. Conclusions

There is apparent confusion among the study participants regarding the use of AP for IE, which is likely due to having multiple guidelines with conflicting recommendations. Less than half of the participants agreed with the change made in the NICE guideline, contributing to resistance in adhering to it. When addressing diverse recommendations, local guidelines, which consider the local clinical and epidemiological conditions, should be the primary point of reference. Nevertheless, we discovered that clinicians who have direct involvement with patients remain highly cognizant of the dental procedures and cardiac conditions that pose an increased risk for IE.

## Figures and Tables

**Table 1:** Questions in section two of the questionnaire.

Content	
(i) Subsection one (three questions): awareness about the 2008 NICE guideline (updated in 2015)	1. Were you aware of the 2008 NICE guideline the first time it was released?
	2. Do you agree with the changes made in the 2008 NICE guideline (updated in 2015)?
	3. Do you think Malaysian specialists and practitioners should adhere to the 2008 NICE guideline (updated in 2015) in relation to no antibiotic prophylaxis prior to invasive dental procedure (i.e., scaling, tooth extraction)?
(ii) Subsection two (eight questions): risk factors involving different types of dental procedures (eight commonly performed dental procedures)	1. What is your opinion about the risks of the following dental procedures in the development of IE?—Routine dental fillings
	2. What is your opinion about the risks of the following dental procedures in the development of IE?—Scaling and polishing
	3. What is your opinion about the risks of the following dental procedures in the development of IE?—Periodontal surgery (gum surgery)
	4. What is your opinion about the risks of the following dental procedures in the development of IE?—Extraction of tooth/teeth
	5. What is your opinion about the risks of the following dental procedures in the development of IE?—Minor surgery for impacted tooth with recent episode of infection
	6. What is your opinion about the risks of the following dental procedures in the development of IE?—Root canal treatment
	7. What is your opinion about the risks of the following dental procedures in the development of IE?—Dental implant surgery
	8. What is your opinion about the risks of the following dental procedures in the development of IE?—Dental extraction in patients with poor oral hygiene
(iii) Subsection three (six questions): different types of cardiac conditions that had previously received antibiotic prophylaxis. We selected the mitral valve stenosis and regurgitation to represent all the known valvular defects	1. Please indicate whether the following cardiac conditions require antibiotic prophylaxis prior to invasive dental procedure (e.g., scaling, tooth extraction, dental implant placement)—Mild mitral stenosis or regurgitation
	2. Please indicate whether the following cardiac conditions require antibiotic prophylaxis prior to invasive dental procedure (e.g., scaling, tooth extraction, dental implant placement)—Moderate mitral stenosis or regurgitation
	3. Please indicate whether the following cardiac conditions require antibiotic prophylaxis prior to invasive dental procedure (e.g., scaling, tooth extraction, dental implant placement)—Severe mitral stenosis or regurgitation
	4. Please indicate whether the following cardiac conditions require antibiotic prophylaxis prior to invasive dental procedure (e.g., scaling, tooth extraction, dental implant placement)—Hypertrophic cardiomyopathy
	5. Please indicate whether the following cardiac conditions require antibiotic prophylaxis prior to invasive dental procedure (e.g., scaling, tooth extraction, dental implant placement)—Previous infective endocarditis
	6. Please indicate whether the following cardiac conditions require antibiotic prophylaxis prior to invasive dental procedure (e.g., scaling, tooth extraction, dental implant placement)—Structural congenital heart disease

**Table 2:** Demographics and specialty details.

Characteristics		Specialty, *n* (%)					Total 277 (100.0)
		OMFS	Cardiologist	Microbiologist	Forensic	FMS	
		55 (100.0)	39 (100.0)	28 (100.0)	47 (100.0)	108 (100.0)	
Age (years)	<40	33 (60.0)	22 (56.4)	19 (67.9)	32 (68.1)	43 (39.8)	149 (53.8)
	40–49	11 (20.0)	14 (35.9)	9 (32.1)	6 (12.8)	36 (33.3)	76 (27.4)
	50–59	9 (16.4)	2 (5.1)	-	6 (12.8)	28 (25.9)	45 (16.2)
	>60	2 (3.6)	1 (2.6)	-	3 (6.4)	1 (0.9)	7 (2.5)
Gender	Male	33 (60.0)	36 (92.3)	5 (17.9)	23 (48.9)	17 (15.7)	114 (41.2)
	Female	22 (40.0)	3 (7.7)	23 (82.1)	24 (51.1)	91 (84.3)	163 (58.8)
Nationality	Malaysian	54 (98.2)	37 (94.9)	28 (100.0)	43 (91.5)	108 (100.0)	270 (97.5)
	Non-Malaysian	1 (1.8)	2 (5.1)	-	4 (8.5)	-	7 (2.5)
Practicing centre	Ministry of Health	37 (67.3)	-	18 (64.3)	41 (87.2)	85 (78.7)	181 (65.3)
	University	14 (25.4)	6 (15.4)	10 (35.7)	5 (10.7)	20 (18.5)	55 (19.8)
	Ministry of Defence	1 (1.8)	-	-	-	-	1 (0.4)
	Private	3 (5.5)	28 (71.8)	-	-	3 (2.8)	34 (12.3)
	Others	-	5 (12.8)	-	1 (2.1)	-	6 (2.2)
Years as a specialist	≤10	40 (72.7)	30 (76.9)	25 (89.3)	37 (78.8)	70 (64.8)	202 (72.9)
	11–20	12 (21.8)	7 (17.9)	3 (10.7)	5 (10.6)	32 (29.6)	59 (21.3)
	>20	3 (5.5)	2 (5.1)	-	5 (10.6)	6 (5.6)	16 (5.8)

FMS, family medicine specialist; OMFS, oral and maxillofacial surgeon.

**Table 3:** Awareness and adherence to the 2008 NICE guideline (updated in 2015).

Questions	Speciality	Total Number of Respondents, (%)			
		Yes	No	Unsure	Total
2.1 Were you aware of the 2008 NICE guideline the first time it was released?	OMFS	43 (78.2)	9 (16.4)	3 (5.5)	55 (100.0)
	FMS	49 (45.4)	47 (43.5)	12 (11.1)	108 (100.0)
	Microbiologist	5 (17.9)	15 (53.6)	8 (28.6)	28 (100.0)
	Forensic Pathologist	11 (23.4)	30 (63.8)	6 (12.8)	47 (100.0)
	Cardiologist	27 (69.2)	9 (23.1)	3 (7.7)	39 (100.0)
	TOTAL	135 (48.7)	110 (39.7)	32 (11.6)	277 (100.0)
2.2 Do you agree with the changes made in the 2008 NICE guideline (updated in 2015)?	OMFS	28 (50.9)	22 (40.0)	5 (9.1)	55 (100.0)
	FMS	43 (39.8)	29 (26.9)	36 (33.3)	108 (100.0)
	Microbiologist	14 (50.0)	5 (17.9)	9 (32.1)	28 (100.0)
	Forensic Pathologist	11 (23.4)	15 (31.9)	21 (44.7)	47 (100.0)
	Cardiologist	21 (53.8)	13 (33.3)	5 (12.8)	39 (100.0)
	TOTAL	117 (42.2)	84 (30.3)	76 (27.4)	277 (100.0)
2.3 Do you think Malaysian specialists and practitioners should adhere to the 2008 NICE guideline (updated in 2015) in relation to no antibiotic prophylaxis prior to invasive dental procedure (i.e., scaling, tooth extraction)?	OMFS	22 (40.0)	30 (54.5)	3 (5.5)	55 (100.0)
	FMS	59 (54.6)	29 (26.9)	20 (18.5)	108 (100.0)
	Microbiologist	18 (64.3)	5 (17.9)	5 (17.9)	28 (100.0)
	Forensic Pathologist	20 (42.6)	10 (21.3)	17 (36.2)	47 (100.0)
	Cardiologist	19 (48.7)	15 (38.5)	5 (12.8)	39 (100.0)
	TOTAL	138 (49.8)	89 (32.1)	50 (18.1)	277 (100.0)

FMS, family medicine specialist; NICE, National Institute for Health and Care Excellence; OMFS, oral and maxillofacial surgeon.

**Table 4:** Association between the specialists and their response to questions 2.1–2.3.

Characteristics	Entire Sample (*n* = 277)			
Specialty	No, *n* (%)	Yes, *n* (%)	Unsure, *n* (%)	*p*
Response to Q2.1				
OMFS	9 (8.2)	43 (31.9)	3 (9.4)	<0.001
Cardiology	9 (8.2)	27 (20.0)	3 (9.4)	
Others	92 (83.6)	65 (48.1)	26 (81.3)	
Response to Q2.2				
OMFS	22 (26.2)	28 (23.9)	5 (6.6)	<0.001
Cardiology	13 (15.5)	21 (17.9)	5 (6.6)	
Others	49 (58.3)	68 (58.1)	66 (86.8)	
Response to Q2.3				
OMFS	30 (33.7)	22 (15.9)	3 (6.0)	<0.001
Cardiology	15 (16.9)	19 (13.8)	5 (10.0)	
Others	44 (49.4)	97 (70.3)	42 (84.0)	

Q2.1: Were you aware of the 2008 NICE guideline the first time it was released? Q2.2: Do you agree with the changes made in the 2008 NICE guideline (updated in 2015)? Q2.3: Do you think Malaysian specialists and practitioners should adhere to the 2008 NICE guideline (updated in 2015) in relation to no antibiotic prophylaxis prior to invasive dental procedure (i.e., scaling, tooth extraction)? FMS, family medicine specialist; NICE, National Institute for Health and Care Excellence; OMFS, oral and maxillofacial surgeon.

**Table 5:** The risk levels of infected endocarditis from selected dental procedures.

Opinion about the Risks of the Following Dental Procedures in the Development of IE	Speciality	Total Number of Respondents (%)				
		Low Risk	Medium Risk	High Risk	Unsure	Total
Routine dental fillings	OMFS	52 (94.6)	1 (1.8)	-	2 (3.6)	55 (100.0)
	FMS	81 (75.0)	21 (19.4)	1 (0.9)	5 (4.7)	108 (100.0)
	Microbiologist	6 (21.5)	17 (60.7)	2 (7.1)	3 (10.7)	28 (100.0)
	Forensic pathologist	27 (57.5)	9 (19.1)	2 (4.3)	9 (19.1)	47 (100.0)
	Cardiologist	32 (82.1)	5 (12.7)	1 (2.6)	1 (2.6)	39 (100.0)
	TOTAL	198 (71.5)	53 (19.1)	6 (2.2)	19 (7.2)	277 (100.0)
Scaling and polishing	OMFS	28 (50.9)	24 (43.6)	3 (5.5)	-	55 (100.0)
	FMS	77 (71.3)	26 (24.1)	1 (0.9)	4 (3.7)	108 (100.0)
	Microbiologist	9 (32.2)	14 (50.0)	3 (10.7)	2 (7.1)	28 (100.0)
	Forensic pathologist	28 (59.6)	9 (19.1)	1 (2.2)	9 (19.1)	47 (100.0)
	Cardiologist	25 (64.1)	13 (33.3)	-	1 (2.6)	39 (100.0)
	TOTAL	167 (60.3)	86 (31.0)	8 (2.9)	16 (5.8)	277 (100.0)
Periodontal surgery (gum surgery)	OMFS	5 (9.1)	31 (56.4)	19 (34.5)	-	55 (100.0)
	FMS	20 (18.5)	53 (49.1)	32 (29.6)	3 (12.8)	108 (100.0)
	Microbiologist	16 (57.2)	7 (25.0)	3 (10.7)	2 (7.1)	28 (100.0)
	Forensic pathologist	7 (14.9)	22 (46.8)	12 (25.5)	6 (12.8)	47 (100.0)
	Cardiologist	11 (28.2)	12 (30.7)	15 (38.5)	1 (2.6)	39 (100.0)
	TOTAL	59 (21.3)	125 (45.1)	81 (29.2)	12 (4.3)	277 (100.0)
Extraction of tooth/teeth	OMFS	7 (12.7)	28 (50.9)	20 (36.4)	-	55 (100.0)
	FMS	37 (34.3)	52 (48.1)	17 (15.7)	2 (1.9)	108 (100.0)
	Microbiologist	12 (42.9)	10 (35.7)	4 (14.3)	2 (7.1)	28 (100.0)
	Forensic pathologist	8 (17.0)	25 (53.2)	10 (21.3)	4 (8.5)	47 (100.0)
	Cardiologist	14 (35.9)	14 (35.9)	10 (25.6)	1 (2.6)	39 (100.0)
	TOTAL	78 (28.2)	129 (46.6)	61 (22.0)	9 (3.2)	277 (100.0)
Minor surgery for impacted tooth with recent episode of infection	OMFS	2 (3.6)	18 (32.7)	34 (61.9)	1 (1.8)	55 (100.0)
	FMS	8 (7.4)	64 (59.4)	32 (29.6)	5 (10.6)	108 (100.0)
	Microbiologist	13 (46.4)	8 (28.6)	5 (17.9)	2 (7.1)	28 (100.0)
	Forensic pathologist	6 (12.8)	15 (31.9)	21 (44.7)	5 (10.6)	47 (100.0)
	Cardiologist	3 (7.7)	18 (46.1)	17 (43.6)	1 (2.6)	39 (100.0)
	TOTAL	32 (11.6)	123 (44.3)	109 (39.4)	13 (4.7)	277 (100.0)
Root canal treatment	OMFS	29 (52.7)	23 (41.8)	3 (5.5)	-	55 (100.0)
	FMS	16 (14.8)	48 (44.4)	38 (35.2)	6 (5.6)	108 (100.0)
	Microbiologist	13 (46.4)	8 (28.6)	5 (17.9)	2 (7.1)	28 (100.0)
	Forensic pathologist	6 (12.8)	20 (42.6)	16 (34.0)	5 (10.6)	47 (100.0)
	Cardiologist	6 (15.4)	17 (43.6)	14 (35.9)	2 (5.1)	39 (100.0)
	TOTAL	70 (25.3)	116 (41.9)	76 (27.4)	15 (5.4)	277 (100.0)
Dental implant surgery	OMFS	4 (7.3)	26 (47.3)	25 (45.4)	-	55 (100.0)
	FMS	13 (12.0)	44 (40.7)	48 (44.5)	3 (2.8)	108 (100.0)
	Microbiologist	14 (50.0)	7 (25.0)	5 (17.9)	2 (7.1)	28 (100.0)
	Forensic pathologist	4 (8.5)	18 (38.3)	17 (36.2)	8 (17.0)	47 (100.0)
	Cardiologist	6 (15.4)	16 (41.0)	15 (38.5)	2 (5.1)	39 (100.0)
	TOTAL	41 (14.8)	111 (40.1)	110 (39.7)	15 (5.4)	277 (100.0)
Dental extraction in patients with poor oral hygiene	OMFS	6 (10.9)	17 (30.9)	32 (58.2)	-	55 (100.0)
	FMS	7 (6.5)	42 (38.9)	57 (52.7)	2 (1.9)	108 (100.0)
	Microbiologist	10 (35.7)	4 (14.3)	12 (42.9)	2 (7.1)	28 (100.0)
	Forensic pathologist	7 (14.9)	9 (19.1)	26 (55.4)	5 (10.6)	47 (100.0)
	Cardiologist	2 (5.1)	12 (30.8)	24 (61.5)	1 (2.6)	39 (100.0)
	TOTAL	32 (11.6)	84 (30.3)	151 (54.5)	10 (3.6)	277 (100.0)

FMS, family medicine specialist; OMFS, oral and maxillofacial surgeon.

**Table 6:** Association between the specialists and their response to the questions in Table 5.

Characteristics	Entire Sample (*n* = 277)			
Specialty	Low Risk, *n* (%)	Medium-to-High Risk, *n* (%)	Unsure, *n* (%)	*p*
Response to risk of dental fillings				
OMFS	52 (26.3)	1 (1.7)	2 (10.0)	<0.001
Cardiology	32 (16.2)	6 (10.2)	1 (5.0)	
Others	114 (57.6)	52 (88.1)	17 (85.0)	
Response to risk of scaling and polishing				
OMFS	28 (16.8)	27 (28.7)	0 (0.0)	0.02
Cardiology	25 (15.0)	13 (13.8)	1 (6.3)	
Others	114 (68.3)	54 (57.4)	15 (93.8)	
Response to risk of periodontal surgery				
OMFS	5 (8.5)	50 (24.3)	0 (0.0)	0.02
Cardiology	11 (18.6)	27 (13.1)	1 (8.3)	
Others	43 (72.9)	129 (62.6)	11 (91.7)	
Response to risk of extraction of teeth				
OMFS	7 (9.0)	48 (25.3)	0 (0.0)	0.01
Cardiology	14 (17.9)	24 (12.6)	1 (11.1)	
Others	57 (73.1)	118 (62.1)	8 (88.9)	
Response to risk of minor oral surgery for an impacted tooth				
OMFS	2 (6.3)	52 (22.4)	1 (7.7)	0.08
Cardiology	3 (9.4)	35 (15.1)	1 (7.7)	
Others	27 (84.4)	145 (62.5)	11 (84.6)	
Response to risk of root canal treatment				
OMFS	29 (41.4)	26 (13.5)	0 (0.0)	<0.001
Cardiology	6 (8.6)	31 (16.1)	2 (13.3)	
Others	35 (50.0)	135 (70.3)	13 (86.7)	
Response to risk of dental implant surgery				
OMFS	4 (9.8)	51 (23.1)	0 (0.0)	0.06
Cardiology	6 (14.6)	31 (14.0)	2 (13.3)	
Others	31 (75.6)	139 (62.9)	13 (86.7)	
Response to risk of dental extraction with poor oral hygiene				
OMFS	6 (18.8)	49 (20.9)	0 (0.0)	0.30
Cardiology	2 (6.3)	36 (15.3)	1 (10.0)	
Others	24 (75.0)	150 (63.8)	9 (90.0)	

FMS, family medicine specialist; OMFS, oral and maxillofacial surgeon.

**Table 7:** Requirement of AP prior to invasive dental procedures in selected cardiac conditions.

Indicate Whether the Following Cardiac Conditions Require AP	Speciality	Total Number of Respondents (%)			
		AP Required	AP Not Required	Unsure	Total
Mild mitral stenosis or regurgitation	OMFS	23 (41.8)	30 (54.5)	2 (3.6)	55 (100.0)
	FMS	35 (32.4)	63 (58.3)	10 (9.3)	108 (100.0)
	Microbiologist	18 (64.3)	6 (21.4)	4 (14.3)	28 (100.0)
	Forensic pathologist	22 (46.8)	14 (29.8)	11 (23.4)	47 (100.0)
	Cardiologist	7 (17.9)	32 (82.1)	-	39 (100.0)
	TOTAL	105 (37.9)	145 (52.3)	27 (9.7)	277 (100.0)
Moderate mitral stenosis or regurgitation	OMFS	34 (61.8)	19 (34.6)	2 (3.6)	55 (100.0)
	FMS	73 (67.6)	32 (29.6)	3 (2.8)	108 (100.0)
	Microbiologist	23 (82.1)	3 (10.8)	2 (7.1)	28 (100.0)
	Forensic pathologist	31 (66.0)	6 (12.8)	10 (21.2)	47 (100.0)
	Cardiologist	19 (48.7)	19 (48.7)	1 (2.6)	39 (100.0)
	TOTAL	180 (65.0)	79 (28.5)	18 (6.5)	277 (100.0)
Severe mitral stenosis or regurgitation	OMFS	42 (76.4)	9 (16.4)	4 (7.2)	55 (100.0)
	FMS	91 (84.3)	12 (11.1)	5 (4.6)	108 (100.0)
	Microbiologist	25 (89.3)	1 (3.6)	2 (7.1)	28 (100.0)
	Forensic pathologist	38 (80.9)	2 (4.2)	7 (14.9)	47 (100.0)
	Cardiologist	31 (79.5)	6 (15.4)	2 (5.1)	39 (100.0)
	TOTAL	227 (81.9)	30 (10.8)	20 (7.3)	277 (100.0)
Hypertrophic cardiomyopathy	OMFS	22 (40.0)	30 (54.5)	3 (5.5)	55 (100.0)
	FMS	53 (49.1)	46 (42.6)	9 (8.3)	108 (100.0)
	Microbiologist	13 (46.4)	6 (21.4)	9 (32.2)	28 (100.0)
	Forensic pathologist	15 (31.9)	18 (38.3)	14 (29.8)	47 (100.0)
	Cardiologist	14 (35.9)	22 (56.4)	3 (7.7)	39 (100.0)
	TOTAL	117 (42.2)	122 (44.0)	38 (13.8)	277 (100.0)
Previous infective endocarditis	OMFS	51 (92.7)	3 (5.5)	1 (1.8)	55 (100.0)
	FMS	96 (88.9)	9 (8.3)	3 (2.8)	108 (100.0)
	Microbiologist	24 (85.7)	1 (3.6)	3 (10.7)	28 (100.0)
	Forensic pathologist	37 (78.7)	2 (4.3)	8 (17.0)	47 (100.0)
	Cardiologist	36 (92.3)	3 (7.7)	-	39 (100.0)
	TOTAL	244 (88.1)	18 (6.5)	15 (5.4)	277 (100.0)
Structural congenital heart disease	OMFS	34 (61.8)	18 (32.7)	3 (5.5)	55 (100.0)
	FMS	63 (58.3)	39 (36.1)	6 (5.6)	108 (100.0)
	Microbiologist	23 (82.1)	1 (3.6)	4 (14.3)	28 (100.0)
	Forensic pathologist	31 (66.0)	6 (12.8)	10 (21.2)	47 (100.0)
	Cardiologist	33 (84.6)	6 (15.4)	-	39 (100.0)
	TOTAL	184 (66.4)	70 (25.3)	23 (8.3)	277 (100.0)

FMS, family medicine specialist; OMFS, oral and maxillofacial surgeon.

**Table 8:** Association between the specialists and their response to questions in Table 7.

Characteristics	Entire Sample (*n* = 277)			
Specialty	AP Not Required, *n* (%)	AP Required, *n* (%)	Unsure, *n* (%)	*p*
Mild mitral stenosis or regurgitation				
OMFS	30 (20.7)	23 (21.9)	2 (7.4)	<0.001
Cardiology	32 (22.1)	7 (6.7)	0 (0.0)	
Others	83 (57.2)	75 (71.4)	25 (92.6)	
Moderate mitral stenosis or regurgitation				
OMFS	19 (24.1)	34 (18.9)	2 (11.1)	0.01
Cardiology	19 (24.1)	19 (10.6)	1 (5.6)	
Others	41 (51.9)	127 (70.6)	15 (83.3)	
Severe mitral stenosis or regurgitation				
OMFS	9 (30.0)	42 (18.5)	4 (20.0)	0.37
Cardiology	6 (20.0)	31 (13.7)	2 (10.0)	
Others	15 (50.0)	154 (67.8)	14 (70.0)	
Hypertrophic cardiomyopathy				
OMFS	30 (24.6)	22 (18.8)	3 (7.9)	0.03
Cardiology	22 (18.0)	14 (12.0)	3 (7.9)	
Others	70 (57.4)	81 (69.2)	32 (84.2)	
Previous IE				
OMFS	3 (16.7)	51 (20.9)	1 (6.7)	0.26
Cardiology	3 (16.7)	36 (14.8)	0 (0.0)	
Others	12 (66.7)	157 (64.3)	14 (93.3)	

AP, antibiotic prophylaxis; FMS, family medicine specialist; IE, infective endocarditis; OMFS, oral and maxillofacial surgeon.

## Data Availability

The datasets used and/or analyzed during the current study are available from the corresponding author on reasonable request.
